# Data Resource Profile: Cohort and Longitudinal Studies Enhancement Resources (CLOSER)

**DOI:** 10.1093/ije/dyz004

**Published:** 2019-02-20

**Authors:** Dara O’Neill, Michaela Benzeval, Andy Boyd, Lisa Calderwood, Cyrus Cooper, Louise Corti, Elaine Dennison, Emla Fitzsimons, Alissa Goodman, Rebecca Hardy, Hazel Inskip, Lynn Molloy, Amanda Sacker, Allan Sudlow, Alice Sullivan, Alison Park

**Affiliations:** 1 CLOSER, UCL Institute of Education, University College London, London, UK; 2 Institute for Social and Economic Research, University of Essex, Colchester, UK; 3 Population Health Sciences, Bristol Medical School, University of Bristol, Bristol, UK; 4 Centre for Longitudinal Studies, UCL Institute of Education, University College London, London, UK; 5 MRC Lifecourse Epidemiology Unit, University of Southampton, Southampton, UK; 6 NIHR Southampton Biomedical Research Centre, University of Southampton and University Hospital Southampton NHS Foundation Trust, Southampton, UK; 7 NIHR Oxford Biomedical Research Centre, University of Oxford, Oxford, UK; 8 UK Data Archive, University of Essex, Colchester, UK; 9 Institute for Fiscal Studies, London, UK; 10 MRC Unit for Lifelong Health and Ageing at UCL, University College London, London, UK; 11 ALSPAC, Population Health Sciences, Bristol Medical School, University of Bristol, Bristol, UK; 12 International Centre for Lifecourse Studies in Society and Health (ICLS), University College London, London, UK; 13 British Library, London, UK; 14 UCL Institute of Education, University College London, London, UK

## Data resource basics

### Context and challenge

The UK has a long history of longitudinal research. The first national birth cohort study was set up in 1946,^1^ and by 2014, one in 30 UK residents were participants in a cohort study.^2^ The first UK household panel study commenced in 1991. As the number of longitudinal studies has increased, the utility of cross-study research has become ever more apparent. In isolation, longitudinal studies can help assess trends and changes among the same individuals over time, but the collation and comparison of data from across studies can also allow researchers to track, quantify and validate changing characteristics at the population level and across generations. Such collation also provides scope for the replication of analyses and, through increased statistical power, helps researchers to investigate rare events and detect smaller associations. Linkage with administrative datasets can similarly produce new research possibilities. Effective collection, integration and use of longitudinal study data do face challenges however, such as:

- divergences in the construct definitions and measurement approaches used over time, between studies and across disciplines;
- gaps in data coverage, due either to the periodic nature of study sweeps (i.e. data collection waves) or to incomplete responses and participant attrition;
- increased volume of data, potentially making discoverability of specific variables, and particularly longitudinally equivalent variables, more difficult;
- and data harmonization and linkage work being undertaken in isolation or anew, potentially duplicating effort and increasing the risk that unintended variations emerge.

Addressing these challenges requires collaborative effort. Drawing together expertise from different disciplines can equip us to identify new learning opportunities and to establish effective tools and standards for facilitating and improving longitudinal research, both within and across individual studies. It also provides a basis on which to build new inter- and cross-disciplinary partnerships, enabling the exchange of both knowledge and skills.

### The origins and aims of CLOSER

The Cohort and Longitudinal Studies Enhancement Resources (CLOSER) consortium was established in October 2012, under the leadership of Professor Jane Elliott, and subsequently Professor Alison Park. The consortium was founded in response to the growing need to foster the integration, enhancement and use of longitudinal data. CLOSER is based at the UCL Institute of Education, UK, and its partners include eight UK longitudinal studies, chosen to reflect a range of longitudinal studies across the biomedical and social sciences domains. Oversight and support for the work undertaken by CLOSER is provided by both a five-member Executive Group and a consortium-wide Leadership Team. The studies within the consortium are listed below, along with their participant numbers at the initial wave of data collection:

- the Hertfordshire Cohort Study (HCS),^3^ a cohort of 3225 men and women born between 1931 and 1939 in the UK county of Hertfordshire, who have been studied across eight sweeps of data collection to date, with the first sweep occurring when participants were at a mean age of 66;
- the Medical Research Council (MRC) National Survey of Health and Development (NSHD),^1^^,^^4^^,^^5^ a nationally representative birth cohort comprising 5362 men and women born in Britain (England, Scotland or Wales) in 1946, with 25 sweeps of data collection completed to date including an initial sweep at birth;
- the 1958 National Child Development Study (NCDS),^6^ a birth cohort comprising 17 415 men and women born in Britain during a single week in 1958, with 11 data collection sweeps completed to date including an initial sweep at birth;
- the 1970 British Cohort Study (BCS70),^7^ a birth cohort comprising 17 198 people born in Britain during a single week in 1970, with the first sweep occurring at birth followed by a further nine sweeps to date;
- the Avon Longitudinal Study of Parents and Children (ALSPAC)^8^^,^^9^ comprises 14 500 men and women born in the former UK county of Avon in 1991-92, as well as their parents and own children; they have undergone 32 data sweeps, their parents have undergone 23 sweeps and the next generation of children have undergone 24 sweeps to date;
- the Southampton Women’s Survey (SWS),^10^ a birth cohort comprising 3158 children born to a sample of 12 583 women who had been recruited before conception (at ages 20-34 years) between 1998 and 2002 in Southampton, England, with four sweeps of data collection before birth, eight completed from birth onwards and a further sweep currently under way with completion expected in 2020;
- the Millennium Cohort Study (MCS),^11^^,^^12^ a nationally representative birth cohort comprising 19 517 children born in the UK (Britain and Northern Ireland) during 2000-02, who have been assessed across six sweeps to date with the first sweep occurring at nine months of age and a further seventh sweep currently underway with completion expected in early 2019;
- Understanding Society: the UK Household Longitudinal Study (UKHLS),^13^ a panel survey comprising 39 802 households across the UK, whose members have been interviewed annually since 2009-10, with eight sweeps completed to date. All members of the household are part of the sample, with parents responding on behalf of any participants aged under 10. This study incorporates 8000 households from the British Household Panel Survey, which began in 1991 and comprised 18 data collection sweeps.

The consortium additionally includes the UK Data Service and the British Library.

CLOSER has five areas of work:

- Data discoverability: given the breadth of social and biomedical data that have been collected over the past three-quarters of a century by UK longitudinal studies, finding specific variables and information about their mode of collection can be challenging due to the volume of data involved and the changing data collection practices. CLOSER works to ensure such information is more easily indexed and searchable across studies as well as across time.
- Data harmonization: CLOSER works to extend the comparability and compatibility of data across longitudinal studies through a series of work packages that, in addition to addressing specific scientific questions, are tasked with systematically identifying equivalent measurements across studies. The work culminates in the creation and dissemination of harmonized datasets for continued research usage.
- Data linkage: CLOSER aims to further extend the quality and scope of longitudinal study data by developing resources to facilitate the linkage of administrative datasets to these study data.
- Impact: CLOSER undertakes a range of activities to improve the visibility of longitudinal data and evidence among practitioners, policy makers, parliamentarians and third sector organizations. CLOSER also draws upon the strength of its members to advocate for a more conducive landscape for longitudinal research. These efforts particularly aim to help drive the development of legislation that facilitates research, or they involve working with regulators and key stakeholders to help promote understanding and effective implementation of existing legislation.
- Training and knowledge exchange: CLOSER draws upon its experience and that of its study partners to develop resources focused on building professional and research capacity and skills in the management, conduct and analysis of longitudinal studies.

Underpinning these five branches of work is CLOSER’s intention to encourage best practice in longitudinal research through the development and dissemination of guidance on effective cross-study research strategies (including through harmonization and linkage work packages, more details of which are provided below), the fostering of interdisciplinary research work and networking activities, and the provision of diverse resources and opportunities for professional development and capacity building. CLOSER’s five areas of work align with the research priorities identified in the Economic and Social Research Council’s (ESRC’s) recent Longitudinal Studies Strategic Review,^14^ which identifies CLOSER as an important resource for longitudinal studies in the UK.

## Data collected

### Measures and data enhancements

**Figure 1.**
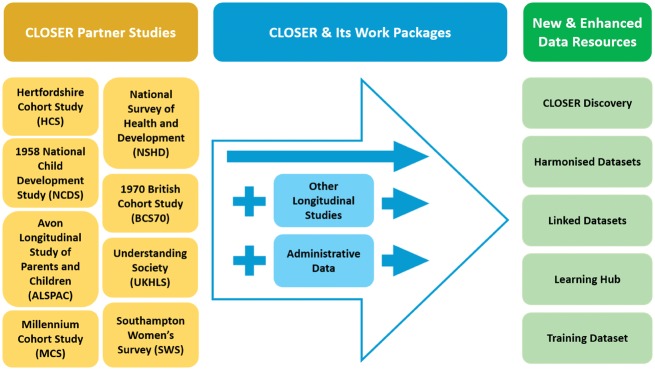
CLOSER’s process of data resource development.

The data which form the basis of CLOSER’s work come from over 80 000 participants in seven UK birth cohort studies and approximately 100 000 members of almost 40 000 households partaking in a UK panel survey. These data have been collected using self-report questionnaires, interviews and clinical assessments, capturing participants’ characteristics throughout their life course and across multiple generations. These data and detailed information on their collection have been sourced by CLOSER and used in the development of new and enhanced resources, as outlined in Figure 1 and discussed in the following sections.

#### Metadata collation and enhancement

Metadata, which have widely been defined as ‘data about data’,^15^ are any information that describes the provenance, format, and meaning of data. The completeness and accuracy of metadata documentation is key in encouraging data re-use, study reproducibility^16^ and the valid interpretation of research findings. Without appropriate documentation, cross-study data integration efforts are also greatly limited.

CLOSER has collated and enhanced metadata from each of its partner studies, to help researchers identify relevant variables across these different sources and to provide comprehensive contextual information to facilitate their use. These metadata have been catalogued in detail according to the Data Documentation Initiative Lifecycle (DDI-L) standard for the documentation of observational measurements.^17^ This provides advantages in terms of enabling cross-cohort comparisons, improving efficiencies in software development through the adoption of an existing data specification framework, and the potential to transform these formatted metadata efficiently to other standards.

The metadata comprise three distinct elements: (i) descriptive metadata for each of the studies; (ii) a CLOSER harmonized ontology which enables the grouping of data/metadata into topic areas; and (iii) structural metadata with associated intra- and inter-study cross-referencing of comparable measures.

**Figure 2.**
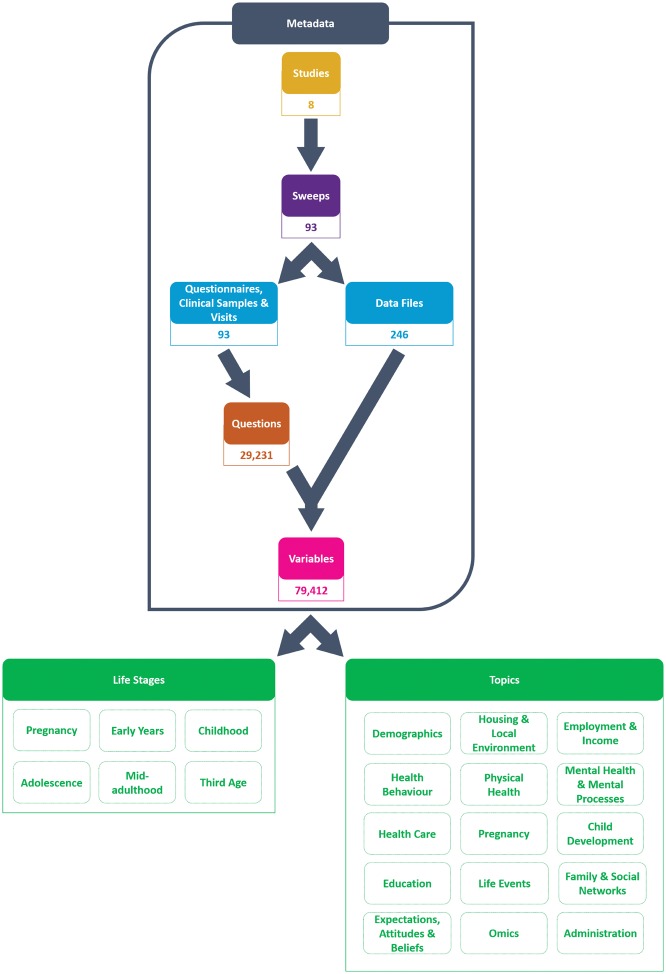
CLOSER Discovery: current coverage and content.

The assembled metadata are made publicly and freely available via an online repository, called CLOSER Discovery, that enables users to locate and explore study questions and variables via text search and filter functions. The repository provides descriptive statistics on the available data for each variable, as well as information on its lineage (such as the study, sweep and questionnaire/data file sources). Figure 2 illustrates the hierarchical process by which the metadata are collated.

Currently, Discovery comprises information sourced from 93 sweeps of the eight CLOSER partner studies, with 79 412 study variables documented to date. These metadata are categorized according to the period of life they cover and the research topics to which they apply (also illustrated in Figure 2). The repository continues to expand as studies conduct new sweeps, and studies outside the CLOSER consortium are also now being added. The first of these is the Whitehall II occupational cohort, a study of 10 308 British civil servants which commenced in 1985.^18^ CLOSER has also recently published a catalogue of the extensive biomarker data collected by CLOSER’s partner studies,^19^ supplementing the utility of Discovery. This provides additional guidance on definition, measurement and interpretation of biomarkers drawn from blood, urine and saliva samples. An overview of the genetic data available from the CLOSER studies is available on the CLOSER website.

CLOSER’s comprehensive documentation of the diverse information collected by UK longitudinal studies helps researchers to effectively locate, use and interpret the large volumes of participant data available. The detailed archiving can assist study coordinators seeking to ensure that backward equivalence is achieved in future study sweeps. Similarly, given the detailed cross-study coverage of variables and the data they include that is offered by CLOSER’s metadata resources, these resources are of particular relevance to data harmonization and linkage efforts.

#### Harmonized variables/datasets

Whereas multiple techniques are available for the joint analysis of data from different studies, such as aggregate data meta-analysis, identifying equivalence in individual-level data further increases the data’s utility and analytical possibilities.^20^ However, this is made complex by the considerable differences in topic coverage and assessment tools that exist both between and within studies, reflecting developments in understanding and assessment practices over time. These variations require recognition and accommodation in any retrospective data harmonization attempt. Harmonization strategies themselves can differ between research groups, and there is consequently a need to better coordinate the standardization and integration of participant data across studies.^21^ Once retrospective harmonization has been carried out, this can help provide clarity regarding concepts and instruments and thus encourage prospective harmonization in future sweeps of data collection.

**Figure 3.**
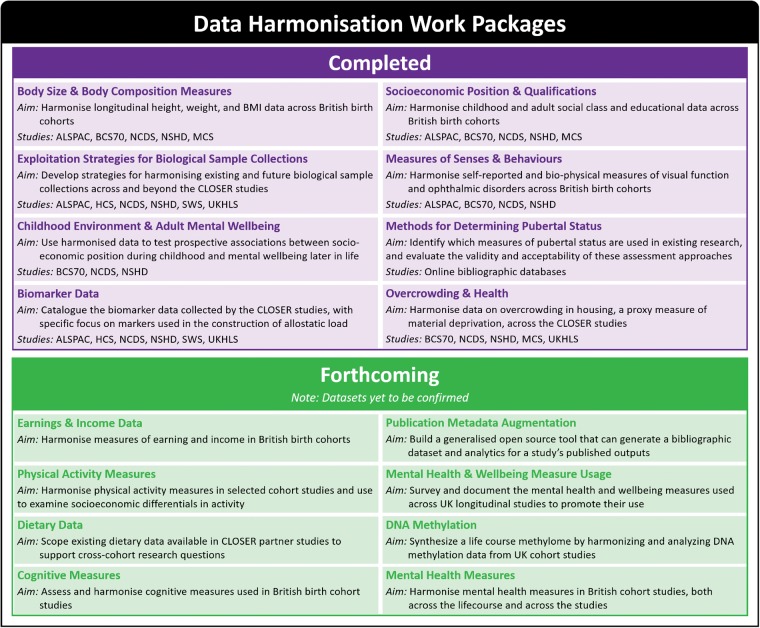
CLOSER data harmonization work packages.

The CLOSER consortium is addressing the challenges of retrospective harmonization by developing harmonization guides and datasets for wider research usage. Eight separate work packages, covering a broad array of applied topics and using a selection of the different CLOSER studies, have been completed to date (see Figure 3). The work packages have each documented the decision-making process involved in the harmonization, including sample selection, data cleaning, and potential limitations. This information is provided in user guides that accompany all harmonized dataset releases. Before release, pseudonymization of the datasets is performed to ensure participant confidentiality across the data management and sharing process. Two sets of harmonized data have been made available via the UK Data Service, with more releases forthcoming. These datasets cover more than six decades of assessment, and enable researchers to examine how diverse biomedical and social characteristics of the UK population have changed across this period. Additional detail on these harmonization work packages, including information on the harmonization approaches used and descriptions of the variables derived, is available as Supplementary data at *IJE* online.

Eight more harmonization work packages are in progress. These cover additional research areas, including dietary data, physical activity measures and DNA methylation data (see Figure 3). Upon completion, datasets and other resources produced from this work will be made available by CLOSER. The work undertaken by CLOSER received ethical approval from the UCL Institute of Education (FPS-447-CLOSER).

#### Data linkage

Linking data held within administrative systems to the data collected by longitudinal studies can enhance the analytical potential of both forms of data, by allowing researchers to combine the rich and varied data collected by longitudinal studies with the detail offered by administrative data.^22^ Linkage can potentially reduce the data collection load on study participants in certain areas of study,^23^ and can help further clarify the relevance of research outputs to decisions on policy and service provision.^24^ Linkage can also enable the cross-validation of self-reported and administrative data^25^^,^^26^ and help address data incompleteness and sampling biases.^27^^,^^28^ However there are a number of practical obstacles to data linkage within the UK, reflecting a range of legal, ethical and social constraints,^29^ with different data sources having different access requirements and restrictions.^23^ Identifying and adopting appropriate strategies for obtaining consent and approval is key.^30^^,^^31^

**Figure 4.**
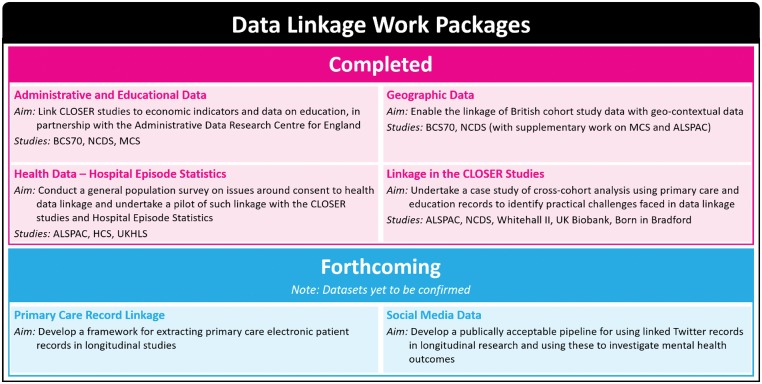
CLOSER data linkage work packages.

CLOSER has coordinated a series of work packages to help improve access to such linked data and promote good practice in this area. These have examined and undertaken the linkage of administrative datasets to longitudinal studies, covering a range of research areas (see Figure 4) and complementing other linkage work being undertaken by the individual CLOSER studies. An awareness of the risk and impact of linkage error and bias has informed this work.

Studies seeking to link centralized health care records to longitudinal research data face several challenges. In recognition of this, CLOSER has worked in tandem with its study partners to develop resources documenting mechanisms by which approval for such linkage can be achieved.^32^

CLOSER has reviewed the scope and potential of geographical variables which could be linked to UK longitudinal data.^33^ It has also expanded the number of studies with geo-coded participant address data (geo-coding to location at a postcode level) in order to facilitate linkage to associated contextual identifiers such as electoral, health and census geographies. This improves researchers’ ability to conduct multi-level modelling, to evaluate changes in geographical characteristics across different time periods and to link additional natural and social environmental data to study data (e.g. pollution exposures, green space provision, neighbourhood quality indices). Further information on the diverse outputs from CLOSER’s data linkage work packages, including information on the linkage methodologies used, is available as Supplementary data at *IJE* online.

Two new data linkage work packages are also now planned, looking at linkage of primary care electronic patient records and social media data (see Figure 4). Their outputs will also be released via the CLOSER website. CLOSER is also continuing to augment its work on improving linkage practice through engagement with data owners and key stakeholders in the UK.

#### Training and knowledge exchange

CLOSER develops a range of resources for training, capacity building and knowledge exchange. These focus particularly on building professional and research capacity and skills in the management, conduct and analysis of longitudinal studies. They typically take the form of workshops and/or resource reports, with relevant examples including a workshop and report on new technologies for health-related data capture in longitudinal studies^34^ as well as a report on NHS Numbers (patient identifiers) and key features of their use with regard to longitudinal studies.^35^ CLOSER has recently run a workshop on the opportunities and challenges of creating and using harmonized datasets, including examples of research undertaken with the CLOSER harmonized datasets described above and the constraints they encountered in making data from different sources more comparable.^36^ To help disseminate the learning from such events and encourage wider knowledge exchange, the materials from these workshops are made available for access and reference via the CLOSER website.

CLOSER has also produced a data resource for educational use. Using NCDS data, CLOSER has derived a cleaned and pseudonymized teaching dataset for students and educators. This dataset comprises 89 variables assessed across eight study waves from 1958 through to 2008. Information on the dataset is available via the CLOSER Learning Hub, an educational platform developed by the consortium. This platform provides training materials for students and educators which introduce the fundamentals of longitudinal research. Using actual data and published study outputs, the Learning Hub offers instruction on terminology, design issues and analytical methods. The platform has evidence sections dedicated to reviewing specific research areas and papers in detail. The site also provides statistical training exercises specific to the teaching dataset, with answer sheets provided which enable learners to appraise their work.

## Data resource use

### Harmonized anthropometric and socioeconomic measures

CLOSER’s data resources have been used in several research projects to date, with more forthcoming as the current work packages conclude. An example is the cross-study research that has been undertaken on body size and composition measures, part of CLOSER’s data harmonization efforts. This harmonization involved the integration of data from 56 425 participants across five cohort studies within the CLOSER consortium. The resultant datasets provide participant weight, height and body mass index (BMI) variables, alongside information on measurement method (self-report or directly assessed), units used (imperial or metric) and measurement precision. The datasets also include a cohort study identifier, a pseudonymized participant code, and demographic details. Table 1 summarizes the data available for the key harmonized variables, with the number of waves and counts provided for each of the source studies. These datasets have recently been used together in an investigation of obesity trajectories across the life course and whether these differ between older and younger generations of UK residents.^37^

**Table 1.** CLOSER harmonized height, weight and BMI variables: data summary

Study	Number of participants (at earliest wave)	Weight		Height		BMI	
		Number of waves	Ages	Number of waves	Ages	Number of waves	Ages
NSHD	4957	13	Birth to 63	12	2-63	12	2-63
NCDS	15 441	9	Birth to 50	8	7-50	8	7-50
BCS70	13 885	7	Birth to 42	7	5-42	6	10-42
ALSPAC	8665	9	7 to 18	9	7-18	9	7-18
MCS	13 477	6	Birth to 11	4	3-11	4	3-11

These data have also been linked to a second set of CLOSER’s data outputs in which longitudinal measures of socioeconomic position (based on occupational social class) were harmonized across four of the CLOSER partner studies. The variables, studies and participant counts are outlined in Table 2. These socioeconomic data have been used alongside the harmonized anthropometric data in two recent studies evaluating life course changes and generational differences in the association between body size and socioeconomic inequality.^38^^,^^39^

**Table 2.** CLOSER harmonized socioeconomic position (SEP) variables: data summary

Study	Number of participants (at earliest wave)	Childhood SEP		Adulthood SEP	
		Number of waves	Ages	Number of waves	Ages
NSHD	5362	1	11	1	42
NCDS	18 558	1	11	1	42
BCS70	14 791	1	11	1	42
MCS	13 287	1	11	0	N/A

N/A, not available.

The socioeconomic data will be extended in the near future. Data on income, collected at a greater number of study waves than those shown in Table 2, have been harmonized and will be made available to researchers via the CLOSER series’ page on the UK Data Service website, as described in the ‘Data Resource Access’ section.

### CLOSER Discovery

**Figure 5.**
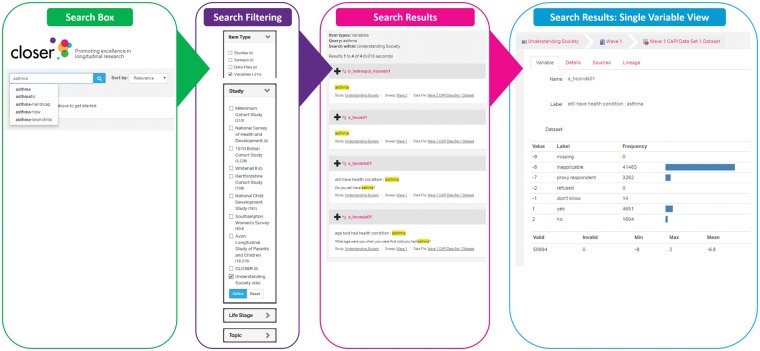
Example of CLOSER Discovery variable search: current asthma status among UKHLS participants with previous asthma diagnosis.

CLOSER Discovery’s interface and search functionality are designed to encourage exploration of the variables collected by participating longitudinal studies and to then provide comprehensive detail on any relevant variables identified. Specific metadata can be retrieved via the Discovery website’s search engine. This tool allows researchers to enter any character string as a search query (e.g. ‘asthma’), and to restrict the search to the study and/or life stage of interest. Alternatively, this information can be accessed by browsing the thematic groupings of the variables as listed on the site’s ‘Explore’ tab. Once researchers locate a variable of interest, the Discovery site provides a detailed metadata summary describing data values, counts and missingness. An example Discovery search process, including the search result, is illustrated in Figure 5. Discovery allows researchers to collate variable lists for future retrieval, and these are retainable across sessions through the creation of a site account. Researchers can also export these variable lists (and their associated metadata) in either a print-ready PDF format or as a DDI XML file for use with external software and to facilitate efficient data extraction in the study repositories.

**Profile in a nutshell**

- CLOSER is a collaboration of multiple British longitudinal studies, as well as the UK Data Service and British Library. This consortium has been established to encourage improvements in the quality of longitudinal research, the data collected in such research and the impact of its findings.
- Drawing upon questionnaire, interview and clinical sample data from seven UK or Britain-based cohort studies and one UK household panel study, CLOSER has developed a range of resources and tools to help facilitate the work of epidemiological and other researchers.
- CLOSER has developed a searchable metadata repository that offers detailed documentation on the data collected across these longitudinal studies, aiding data discoverability [https://discovery.closer.ac.uk/].
- CLOSER has also coordinated a series of work packages on the harmonization and linkage of longitudinal study data, covering a range of demographic, socioeconomic, educational and health domains. Datasets and learning tools have been developed as part of this work, and these resources are made available to researchers and practitioners via the CLOSER website [https://www.closer.ac.uk/] and the UK Data Service [https://discover.ukdataservice.ac.uk/series/? sn=2000111].

CLOSER is currently working to establish equivalency between variables to improve the ease with which researchers can identify related variables across multiple sweeps of a study and between studies. To date, these ‘concordance variables’ have been identified and made available via Discovery for the ALSPAC study. Future work will document concordance variables for all studies listed on the repository.

In addition to aiding data discoverability and guiding researchers in their engagement with study metadata, Discovery is also being used to assess and develop standards for data documentation. CLOSER is actively involved in the DDI Alliance, and work on this resource has been presented at a number of international conferences on metadata management.^40^^,^^41^

## Strengths and weaknesses

By bringing together existing longitudinal studies and sponsoring new research projects that use these data, CLOSER is equipped to identify best practice in longitudinal research and to document solutions to the hurdles faced in the use of such studies’ data. The principal benefits of CLOSER’s work include: (i) the broad, cross-study focus that facilitates collaborative, interdisciplinary endeavours, including support for networking, knowledge/skill exchange, stakeholder engagement (particularly with regard to the policy making community) and advocacy work; (ii) the centralized access to detailed information on the many variables collected across multiple sweeps by different longitudinal studies; (iii) the widened perspective on patterns of generational change offered by the harmonized datasets created by CLOSER and its collaborators; (iv) the increased breadth of insight and potential for improved data validity offered by linking administrative datasets to the CLOSER partner studies; and (v) the sourcing of diverse expertise to generate free-to-access outputs, as well as training tools and workshops. The benefits of CLOSER’s work in these areas were clearly acknowledged in the ESRC’s recent review of the longitudinal study landscape.^14^ CLOSER Discovery in particular is identified in the report as an important aid to researchers seeking information on available study data.

The production of CLOSER’s resources has involved challenges however. CLOSER has worked to integrate data, establish learning tools and improve research impact from across diverse studies, but these studies also have differing aims and objectives, which have influenced their designs and can make their comparisons challenging. The scale of the work undertaken by CLOSER means that its focus has to date primarily been on data collected by its study partners, but efforts are now being made to expand this coverage to new studies. Even as harmonization practices improve, there will continue to be limitations to the extent to which harmonization can be performed for all variables. Where there are sizeable differences in the operationalization of variables or calibration of measurement instruments, it may not be feasible to attempt harmonization. CLOSER is undertaking work in this area, however, to assess the scope and impact of such calibration issues.^42^ All longitudinal study data are vulnerable to participant attrition and data missingness. Linking administrative data to longitudinal studies can address some gaps in coverage, but the data resources that CLOSER generates will still always require consideration of the presence and impact of missing data. Finally, there will always be challenges in securing administrative data linkage, as licensing agreements can vary, information governance policies are subject to change, and access negotiation can be a time- and resource-intensive process. Recognizing this, CLOSER is documenting the challenges faced in data linkage efforts and is publishing guidance for other researchers on how to navigate such difficulties.

## Data resource access

The CLOSER Discovery metadata and data enhancement programme is hosted on the CLOSER website [https://discovery.closer.ac.uk/]. The website includes the detailed filter and search functionality outlined above. Training materials for researchers interested in Discovery are available on the CLOSER site. The data management software that has been developed by CLOSER during the creation of CLOSER Discovery are hosted on the consortium’s GitHub site [https://github.com/CLOSER-Cohorts].

To assist researchers undertaking data integration work such as harmonization or linkage, CLOSER has published resource reports and guidance documents on its website [https://www.closer.ac.uk/resources/]. The site also includes slides and recordings from workshop presentations on these topics. The harmonized datasets produced by CLOSER are made available via the consortium’s series record on the UK Data Service [https://discover.ukdataservice.ac.uk/series/? sn=2000111]. Currently this includes datasets comprising height, weight and BMI measures from five UK longitudinal cohort studies [NCDS, BCS70, MCS, NSHD and ALSPAC] and additional datasets providing harmonized socioeconomic data for four of these studies [BCS70, MCS, NCDS and NSHD]. Data sourced from NCDS, BCS70 and MCS are available under UK Data Service’s End User Licence requiring researchers to complete user and project registration to access the data. For data sourced from ALSPAC and NSHD, Special Licence usage terms apply and, in addition to the requirements of the End User Licence, researchers are required to submit a detailed research application for review before data release. Guidance on accessing the original data from the CLOSER partner studies is provided, alongside details on genetic data availability, on CLOSER’s main website [https://www.closer.ac.uk/how-to-access-the-data].

CLOSER’s training materials can also be accessed via CLOSER’s website, with a specific section dedicated to the CLOSER Learning Hub [https://learning.closer.ac.uk]. This includes links to the CLOSER teaching dataset which is hosted by the UK Data Service. Materials from CLOSER’s previous training events are also made available on the CLOSER website, including content from CLOSER’s recent workshop on cross-study data harmonization [https://www.closer.ac.uk/news-opinion/blog/crossstudy-research-overcoming-obstacles-uncovering-opportunity/].

Further datasets from CLOSER’s harmonization work will also be made available via CLOSER’ series page on the UK Data Service. The geographical identifier data developed as part of CLOSER’s data linkage work are also available from the UK Data Service. Researchers who use CLOSER data resources, including the harmonized datasets, are requested to appropriately cite them in research outputs. Citation guidance is provided with all data downloads.

## Funding

The CLOSER consortium is supported by funding from the Economic and Social Research Council (ESRC) and the Medical Research Council (MRC). An initial grant for this work was awarded to CLOSER in 2012 and extended, by the ESRC, in 2017 (award reference: ES/K000357/1).

**Conflict of interest:** None declared.

## Supplementary Material

dyz004_Supplementary_Data
