# Supplementary material for: Data Resource Profile: Cohort and Longitudinal Studies Enhancement Resources (CLOSER)
Source: Int J Epidemiol. 2019 Feb 20;48(3):675–676i. doi: 10.1093/ije/dyz004 (PMC6659357; doi:10.1093/ije/dyz004)
Supplement: dyz004_Supplementary_Data [file dyz004_supplementary_data.docx]

# Supplementary Materials

## Citation list for work published on the CLOSER data resource

The current list is valid as of August 2018; please check the CLOSER website for the latest publications and resources (<https://www.closer.ac.uk/>).

#### General Journal Articles

Park A. Working with longitudinal data: How CLOSER can help. Soc Res Assoc Res Matters. 2015;3:8.

Kordas K, Park A. European birth cohorts offer insights on environmental factors affecting human development and health. Int J Epidemiol. 2015;44(3):731–34.

Cohort and Longitudinal Studies Enhancement Resources. Getting CLOSER to cohort studies. Soc Res Assoc Res Matters. 2013;3:5.

Wallace SE, Walker NM, Elliott J. Returning findings within longitudinal cohort studies: The 1958 birth cohort as an exemplar. Emerg Themes Epidemiol. 2014;11:10.

#### Harmonisation Journal Articles

Bann D, Johnson W, Li L, Kuh D, Hardy R. Socioeconomic inequalities in body mass index across adulthood: Coordinated analyses of individual participant data from three British birth cohort studies initiated in 1946, 1958 and 1970. PLOS Med. 2017;14(1):e1002214.

Bann D, Johnson W, Li L, Kuh D, Hardy R. Socioeconomic inequalities in childhood and adolescent body-mass index, weight, and height from 1953 to 2015: An analysis of four longitudinal, observational, British birth cohort studies. Lancet Public Heal. Elsevier; 2018;3:e194–203.

Bountziouka V, Cumberland PM, Rahi JS. Trends in visual health inequalities in childhood through associations of visual function with sex and social position across 3 UK birth cohorts. JAMA Ophthalmol. 2017;135(9):954–961.

Bountziouka V, Cumberland PM, Rahi JS. Trends in visual health inequalities in childhood through associations of visual function with sex and social position across 3 UK birth cohorts - Reply. JAMA Ophthalmol. 2018;136(2):223.

Cumberland PM, Bountziouka V, Rahi JS. Impact of varying the definition of myopia on estimates of prevalence and associations with risk factors: Time for an approach that serves research, practice and policy. Br J Ophthalmol. Forthcoming 2018.

Johnson W, Li L, Kuh D, Hardy R. How has the age-related process of overweight or obesity development changed over time? Co-ordinated analyses of individual participant data from five United Kingdom birth cohorts. PLOS Med. 2015;12(5):e1001828.

Wood N, Bann D, Hardy R, et al. Childhood socioeconomic position and adult mental wellbeing: Evidence from four British birth cohort studies. PLOS One. 2017;12(10):e0185798.

#### Harmonisation Resource Reports & Working Papers

Belfield C, Crawford C, Greaves E, Gregg P, Macmillan L. Intergenerational income persistence within families. London, UK: Institute of Fiscal Studies; 2017. Available from: https://www.ifs.org.uk/uploads/publications/wps/WP201711.pdf

Belfield C, van der Erve L. Returns to higher education for women: In the labour market and marriage market. London, UK: Institute of Fiscal Studies; 2018.

Belfield C, van der Erve L. The impact of higher education on the living standards of female graduates. London, UK: Institute of Fiscal Studies; 2018. Available from: https://www.ifs.org.uk/uploads/publications/wps/WP201825.pdf

Baird J, Walker I, Smith C, Inskip H. Review of methods for determining pubertal status and age of onset of puberty in cohort and longitudinal studies. London, UK: CLOSER; 2017. Available from: https://www.closer.ac.uk/wp-content/uploads/Review-of-methods-for-determining-pubetal-status-FULL-FINAL.pdf

Lessof C, Wong A, Hardy R, CLOSER Equipment Comparison Team. Early findings from a randomised repeated-measurements cross-over trial to understand differences in measures of physiological function and physical performance. 5th Panel Survey Methods Workshop; 2016 June 20-21. Berlin, Germany: German Institute for Economic Research; 2016. Available from: https://www.diw.de/documents/dokumentenarchiv/17/diw_01.c.535244.de/psmw2016_lessof%20et%20al_2016_early%20findings.pdf

Rajatileka S, Groom A, Ring S. Development of functional assays in lymphoblastoid cell lines. London, UK: CLOSER; 2017. Available from: https://www.closer.ac.uk/wp-content/uploads/Development-of-functional-assays-in-lymphoblastoid-cell-lines.pdf

Rajatileka S, Groom A, Ring S. Effects of sample processing on the metabolome and proteome. London, UK: CLOSER; 2017. Available from: https://www.closer.ac.uk/wp-content/uploads/Effects-of-sample-processing-on-the-metabolome-and-proteome-1.pdf

Rajatileka S, Groom A, Ring S. Harmonisation of strategies for exploitation of biological sample collections. London, UK: CLOSER; 2017. Available from: https://www.closer.ac.uk/wp-content/uploads/Harmonisation-of-strategies-for-exploitation-of-biological-sample-collections.pdf

Rajatileka S, Groom A, Ring S. Histone methodology development report. London, UK: CLOSER; 2017. Available from: https://www.closer.ac.uk/wp-content/uploads/Histone-Methodology-Development-report-1.pdf

Ruiz M, Benzeval M, Kumari M. A guide to biomarker data in the CLOSER studies: A catalogue across the cohort and longitudinal studies. London, UK: CLOSER; 2017. Available from: https://www.closer.ac.uk/wp-content/uploads/A-guide-to-the-biomarker-data-in-the-CLOSER-studies.pdf

#### Linkage Resource Reports & Journal Articles

Feng Z, Dibben C. A review of resources for geographical variables. London, UK: CLOSER; 2013. Available from: https://www.closer.ac.uk/wp-content/uploads/CLOSER-resource-report-2-geographical-variables-final.pdf

Boyd A, Thomas, R, Macleod, J. NHS Number and the systems used to manage them: An overview for research users. London, UK: CLOSER; 2018. Available from: https://www.closer.ac.uk/wp-content/uploads/CLOSER-NHS-ID-Resource-Report-Apr2018.pdf

Boyd A. NHS Digital: Precedents set. London, UK: CLOSER; 2018. Available from: https://www.closer.ac.uk/wp-content/uploads/CLOSER-NHS-Digital-Precedent-Set-Resource-Report-Apr18.pdf

Boyd A, Cornish R, Johnson L, Simmonds S, Syddall H, et al. Understanding Hospital Episode Statistics (HES). London, UK: CLOSER; 2018. Available from: https://www.closer.ac.uk/wp-content/uploads/CLOSER-Understanding-Hospital-Episode-Statistics-Resource-Report.pdf

Knies G, Burton J. Analysis of four studies in a comparative framework reveals: Health linkage consent rates on British cohort studies higher than on UK household panel surveys. *BMC Med Res Methodol*. 2014;14(1):125.

Parsons S. Mass observation archive: How to combine information with the British birth cohort studies. London, UK: CLOSER; 2013. Available from: https://www.closer.ac.uk/wp-content/uploads/CLOSER-resource-report-1-Mass-Observation-Archive-S-Parsons-May-2013-FINAL.pdf

#### Knowledge Exchange Resource Reports

Stone CJ, Skinner AL. New technology and novel methods for capturing health-related data in longitudinal and cohort studies: Report from a CLOSER workshop. London, UK; 2017. Available from: https://www.closer.ac.uk/wp-content/uploads/New-technology-and-novel-methods-for-capturing-health-related-data.pdf

Jäckle A, Gaia A, Benzeval M. The use of new technologies to measure socio-economic and environmental concepts in longitudinal studies. London, UK; 2018. Available from: https://www.closer.ac.uk/wp-content/uploads/New-technology-and-novel-methods-for-capturing-health-related-data.pdf

Jäckle A, Gaia A, Benzeval M. Mixing modes and measurement methods in longitudinal studies. London, UK: CLOSER; 2017. Available from: https://www.closer.ac.uk/wp-content/uploads/Mixing-modes-and-measurement-methods-in-longitudinal-studies-1.pdf

#### Discovery Resource Reports & Presentations

Kaye J. User needs study for the Uniform Search Platform (USP). London, UK: CLOSER; 2014.

Poynter W, Spiegel J. Protocol development for large-scale metadata archiving using DDI-Lifecycle. *European DDI User Conference; 2014 December 2-3*. London, UK; *2014*.

Poynter W, Spiegel J. Protocol development for large-scale metadata archiving using DDI-Lifecycle. *IASSIST Q*. 2015;39(3):23–29.

Seabrook G. Doing DDI: Operationalising DDI for longitudinal studies. *International Association for Social Science Information Services & Technology; 2015 June 2-5*. Minneapolis, USA; 2015.

Johnson J. Lessons learnt from the development of a DDI-based discovery platform. *European DDI User Conference; 2016 December 6-7*. Cologne, Germany; 2016.

Mills H. Lessons from a large-scale metadata archiving team using DDI-Lifecycle. *European DDI User Conference; 2016 December 6-7*. Cologne, Germany; 2016.

Poynter W. Archivist & Mapper: Simplifying and modernising questionnaire entry. *European DDI User Conference; 2016 December 6-7*. Cologne, Germany; 2016.

Poynter W. CLOSER repository: Modernising longitudinal study management. *European Survey Research Conference; 2017 July 18-21*. Lisbon, Portugal; *2017*.

Mills H. CLOSER Discovery: Past, present and future. *European DDI User Conference; 2017 December 5-6*. Lausanne, Switzerland; *2017*.

Poynter W. An application of search engine topics through the use of DDI-3.2 concepts. *European DDI User Conference; 2017 December 5-6*. Lausanne, Switzerland; *2017*.

## Harmonisation work packages: Additional detail

| Work package | Aim | Source studies | Research lead | Outputs: Datafiles and resource reports | Overview |
| --- | --- | --- | --- | --- | --- |
| Body Size & Body Composition Measures | Harmonise longitudinal height, weight, and BMI data across British birth cohorts | ◦ ALSPAC ◦ BCS70 ◦ NCDS ◦ NSHD ◦ MCS | Rebecca Hardy, UCL | ◦ Harmonised NCDS, BCS70 & MCS data: *http://doi.org/10.5255/UKDA-SN-8207-1*  ◦ Harmonised ALSPAC data: *http://doi.org/10.5255/UKDA-SN-8208-1*  ◦ Harmonised NSHD data: *http://doi.org/10.5255/UKDA-SN-8209-1* | This project explored how rates of overweight and obesity are changing across generations. Weights and heights were converted to kilograms and metres, respectively, when originally recorded on imperial scales. Measured data at age 16 years in the BCS70 were augmented with 2,353 self-reported weights and 2,309 self-reported heights at the same age to maximise the amount of available information. Further, measured data at age 44 years in the NCDS were augmented with 12 observations of self-reported weight greater than 150 kg, in an attempt to retrieve information from the upper end of the distribution that appeared to have been removed by the employment of a cut-off during data entry or cleaning. 9,063 missing observations of height at age 50 in NCDS were filled in with observations of height from the sweep at age 44 years. The same strategy had already been applied to study derived variables of height at ages 34 (filled in using age 30 year data) and 42 years (filled in using age 30 or 34 year data) in BCS70. Where variables of decimal age at assessment were not available, they were computed from existing age variables or as the difference between date of birth and date of assessment. Participants who were missing decimal age were assigned the mean value for that cohort at that sweep. A standardised data cleaning protocol was applied. The research utility of this dataset has been demonstrated through a number of recent journal articles, as referenced in the above bibliography.  The published dataset includes harmonised variables from multiple data sweeps across the life-course for each of the 5 longitudinal studies, covering height, weight, and body-mass index measurements, as well as detail on measurement method, scale, and precision.  This overview of the harmonisation process and derived variables is sourced from the dataset user guide: Hardy R, Johnson J, Park A. CLOSER Work Package 1: Harmonised Height, Weight and BMI User Guide. London, UK: CLOSER, 2016. The guide offers further detail on the sample selection and data cleaning involved, and is available alongside the datasets on the UK Data Service. |
| Socioeconomic Position & Qualifications | Harmonise childhood and adult social class and educational data across British birth cohorts | ◦ ALSPAC ◦ BCS70 ◦ NCDS ◦ NSHD ◦ MCS | Claire Crawford & Chris Belfield, Institute for Fiscal Studies | ◦ Harmonised NSHD data: *http://doi.org/10.5255/UKDA-SN-8308-1*  ◦ Harmonised NCDS data: *http://doi.org/10.5255/UKDA-SN-8307-1*  ◦ Harmonised BCS70 data: *http://doi.org/10.5255/UKDA-SN-8305-1*  ◦ Harmonised MCS data: *http://doi.org/10.5255/UKDA-SN-8306-1*  ◦ Harmonised ALSPAC data: In preparation for release on UK Data Service  ◦ Harmonised datasets covering educational achievement and qualifications are also in preparation for release for NCDS, BCS70, and MCS. | This project has included the harmonisation of socio-economic status data across different generations using five UK longitudinal studies. A range of possible socio-economic measures were considered before choosing one around which all five studies could be harmonised. The most difficult problem was the wide historical range of the starting-years for the five surveys, from 1946 (NSHD) to 2000/01 (MCS). During this 55-year period, different official methods of occupational classification have superseded each other every decade, as have many derived measures of socio-economic status.  For the purposes of this project, the decision was made to standardise on RG Class (1990 version), which stood chronologically in the middle of the period 1946-2016, and was the measure to which, with some minor exceptions, the measures of all six studies could be converted. Given that the six studies surveyed their respective participants at varying ages, a decision was taken to limit the harmonisation of social class to just one childhood time-point (for parent’s occupation, nearest to age 10/11) and one adult time-point (nearest to age 42). For NCDS and BCS70 at age 10/11 there were a small number of cases where the desired indicator of parental socioeconomic class was not available. A value was derived for these from parallel variables available in the datasets and a flag was provided to identify such cases. In the case of MCS, the cohort are still not old enough to have an adult time-point.  The published dataset consequently comprises harmonised variables for parental social class when the study participant was aged 10-11 and the participants' own social class when aged 42-43.  This overview of the harmonisation process and output is sourced from the dataset user guide which includes further detail and is available alongside the datasets on the UK Data Service: Dodgeon B, Morris T, Crawford C, Parsons S, Vignoles A, Oldfield Z. CLOSER Work Package 2: Harmonised Socio-Economic Measures User Guide. London, UK: CLOSER, 2018. |
| Exploitation Strategies for Biological Sample Collections | Develop strategies for harmonising existing and future biological sample collections across and beyond the CLOSER studies | ◦ ALSPAC ◦ HCS ◦ NCDS ◦ NSHD ◦ SWS ◦ UKHLS | Susan Ring, University of Bristol | ◦ Harmonisation of strategies for exploitation of biological sample collections: *https://www.closer.ac.uk/wp-content/uploads/CLOSER-resource-Harmonisation-of-strategies-for-exploitation-of-biological-sample.pdf*  ◦ Histone methodology development report: *https://www.closer.ac.uk/wp-content/uploads/CLOSER-resource-Histone-methodology-development-report.pdf*  ◦ Effects of sample processing on the metabolome and proteome: *https://www.closer.ac.uk/wp-content/uploads/CLOSER-resource-Effects-of-sample-processing-on-the-metabolome-and-proteome.pdf*  ◦ Development of functional assays in lymphoblastoid cell lines: *https://www.closer.ac.uk/wp-content/uploads/CLOSER-resource-Development-of-functional-assays-in-lymphoblastoid-cell-lines.pdf* | Biological samples such as blood, urine and saliva are routinely collected by many cohort studies and used to explore questions around health and biological changes over time. Different methods have been used for processing, storing and analysing these samples. This project explored how the collection, processing and storage conditions of biological samples could be made more consistent across longitudinal studies. It also summarised the existing biological samples collected by CLOSER studies, facilitating the cross-study usage of such data.  In addition, the team undertook a small pilot study which tested new laboratory methods for processing blood samples. The project produced new documentation to help researchers to improve methods and standards for the collection, processing and storage of biological samples used in longitudinal studies. |
| Measures of Senses & Behaviours | Harmonise self-reported and bio-physical measures of visual function and ophthalmic disorders across British birth cohorts | ◦ ALSPAC ◦ BCS70 ◦ NCDS ◦ NSHD | Jugnoo Rahi, UCL | ◦ Data in preparation for release on UK Data Service | In the three national birth cohorts, (NSHD, NCDS and BCS70), measurements in each eye were taken during childhood by a school doctor using the conventional Snellen charts, composed of block capital letters without serifs. Visual acuity values across the cohorts range from “6/4” to “more than 6/60”. A mapping framework was devised to harmonise the labelling of comparable values across the cohorts. Using distance acuity in each eye, the harmonisation team assigned individuals to one of six mutually exclusive categories, ranging from normal vision to visual impairment, severe visual impairment or blindness, extending the World Health Organization taxonomy of visual impairment to include vision loss at a level recognised to affect personal and social life.  In ALSPAC, visual acuity data on visual acuity were not available but information was collected on refractive error . Refraction status was assessed using an autorefractor that provided refraction data, including sphere and cylinder (astigmatism) measures. Spherical equivalent is a measure of the power of a lens prescription required to obtain normal distance vision and is calculated using a standard formula, incorporating the values of sphere and plus cylinder. The average of spherical equivalent from left and right eye is conventionally used for the calculation of mean spherical equivalent, and the estimation of the refractive error frequency in a population. Cut-offs were adopted to define the refractive error status, based on either the spherical equivalent for each eye or the mean spherical equivalent for the prevalence of myopia.  The harmonised dataset produced through this work is due to be published and comprises data on categories of distance visual acuity at ages 15-16 for NSHD, NCDS, and BCS70, as well as data on refractive error and categories of refractive error at age 15 from ALSPAC.  This description of the dataset is sourced from the user guide that will be made available alongside the dataset when it is published on the UK Data Service. |
| Childhood Environment & Adult Mental Wellbeing | Use harmonised data to test prospective associations between socio-economic position during childhood and mental wellbeing later in life | ◦ BCS70 ◦ NCDS ◦ NSHD | Mai Stafford, The Health Foundation | ◦ Data in preparation for release on UK Data Service | This harmonisation project used the following approach in making the measures of the childhood environment more comparable across the studies. Firstly, the literature on childhood risk factors for poor mental health and mental wellbeing in adulthood was used to identify relevant domains of the childhood environment. Five domains of the childhood environment were identified: socio-economic, child rearing and parenting, family instability, parental health, and antenatal/perinatal problems. The harmonisation team considered that these captured circumstances that were “external” to the child. Factors such as childhood cognition, illness and mental health may be influenced by earlier childhood circumstances and are arguably on the explanatory pathway linking the childhood environment to later mental wellbeing and they were not the main focus of this harmonisation project.  Once the childhood environment domains had been identified, the most relevant variables were found for each study. Recognising the limits of retrospective harmonisation across all these childhood environment domains, the project team aimed to identify variables that capture similar constructs for each of these domains and take a coordinated analytical approach to maximise comparability across the studies.  When assessing comparability of measures across the studies, the team looked at who answered the question (i.e. a cohort member, a parent, a teacher or a health visitor), the age of the cohort member when the measure was collected, as well as the question wording. In most instances, the team used the original questions, but they also used previously derived variables and did not go back to the original data if the derived measure was suitable. Variables were derived which had the same categories across the studies, where possible. When data were collected on the same measure at multiple sweeps in childhood, the team derived the mean across the sweeps or counted the number of sweeps in a particular level of exposure.  The data due to be published will include harmonised variables covering a number of domains, including family socio-economic position, child rearing/parenting, family instability, and parental health. A harmonised measure of wellbeing in later life will also be included, with this covering a cross-study age range of 42-64.  This description of the work package is sourced from the dataset user guide, which will be made available alongside the data when they are published on the UK Data Service. |
| Methods for Determining Pubertal Status | Identify which measures of pubertal status are used in existing research, and evaluate the validity and acceptability of these assessment approaches | ◦ Online bibliographic databases | Janis Baird & Hazel Inskip, University of Southampton | ◦ Review of methods for determining pubertal status and age of onset of puberty in cohort and longitudinal studies: *https://www.closer.ac.uk/wp-content/uploads/CLOSER-resource-Review-of-methods-for-determining-pubertal-status-and-age-of-onset-of-puberty-in-cohort-and-longitudinal-studies.pdf* | Measuring the stage at which young people experience puberty is valuable in the assessment of various health outcomes in adolescents, especially as the hormonal changes associated with puberty can impact on both physical and mental wellbeing. This research project identified and assessed measures used to determine pubertal status, identifying similarities among methods that would allow for harmonisation across cohort datasets.  It also consulted a children’s patient and public involvement (PPI) committee to assess the acceptability of various approaches to measuring pubertal status, and identify what young people might consider as barriers to using these assessments. A report has been published to help researchers assess the validity of measures used to determine pubertal status in cohort and other studies around the world. |
| Biomarker Data | Catalogue the biomarker data collected by the CLOSER studies, with specific focus on markers used in the construction of allostatic load | ◦ ALSPAC ◦ HCS ◦ NCDS ◦ NSHD ◦ SWS ◦ UKHLS | Meena Kumari, University of Essex | ◦ A guide to biomarker data in the CLOSER studies: *https://www.closer.ac.uk/wp-content/uploads/CLOSER-resource-A-guide-to-the-biomarker-data-in-the-CLOSER-studies.pdf* | This project produced a comparative catalogue of the biomarkers available across the CLOSER studies. A biomarker is a measurable biological characteristic or medical sign that is judged to be an indicator of normal biological processes or detrimental biological processes and certain diseases – for example, cholesterol levels are a biomarker and risk indicator for heart disease.  The catalogue focuses on biomarkers that can be extracted from samples of blood, saliva or urine, and includes data from six CLOSER studies. The project team looked specifically at which biological markers are typically included when calculating allostatic load, which is the term used to describe the biological ‘wear and tear’ associated with the body’s response to chronic or repeated episodes of stress. The catalogue is made available on the CLOSER website. |
| Overcrowding & Health | Harmonise data on overcrowding in housing, a proxy measure of material deprivation, across the CLOSER studies | ◦ BCS70 ◦ NCDS ◦ NSHD ◦ MCS ◦ UKHLS | Noriko Cable, UCL | ◦ Data in preparation for release on UK Data Service | This project investigated household overcrowding and its implications across generations, by producing comparable measures of overcrowding from household information collected by five CLOSER studies. Overcrowding has long been treated as a proxy indicator for material deprivation, but different approaches have been used to determine overcrowded households.  In this project, three overcrowding measures have been derived for each of the studies: persons per room, the bedroom standard, the modified bedroom standard. The persons per room approach divides numbers of rooms, excluding kitchen or bathrooms by the number of household occupants to indicate overcrowding. The bedroom standard has been defined as: a bedroom for each couple; a bedroom for each single person age 21 and over; a shared bedroom for each pair of same-sex adolescents (10 – 20 years); a shared bedroom for each pair of same or opposite sex children (under 10 years old); a shared bedroom for a remaining unpaired same-sex adolescent and child; a separate bedroom for each remaining adolescent or child. If the standard number of bedrooms is greater than the actual number of bedrooms, the home is considered overcrowded. Finally, the modified bedroom standard applies the same rules but with children under 8 years old, adolescents 8 to 17 years old, and adults 18 years old and over. These variables have been derived from housing information across multiple waves for each of the studies. The validity of the three measures was tested against existing SES indicators for a subset of the data and a paper on this work is being prepared for publication.  Additional detail will also be provided in the dataset user guide which will be made available alongside the data when they are published on the UK Data Service. |

## Data linkage work packages: Additional detail

| Work package | Aim | Source studies | Research lead | Outputs: Datafiles and resource reports | Overview |
| --- | --- | --- | --- | --- | --- |
| Administrative and Educational Data | Link CLOSER to economic indicators and data on education, in partnership with the Administrative Data Research Centre for England | ▫ BCS70 ▫ NCDS ▫ MCS | Alissa Goodman & Lorraine Dearden, UCL | ▫ Consistent Schools Database: *Researchers interested in using the CSD can request the dataset by emailing closer@ucl.ac.uk, giving your name and place of work, and explaining what research you intend to carry out using the data.* | In the UK, the government is the main collector and custodian of administrative records; examples include data collected regarding tax and benefits, education and health. This project worked towards linking data from specific cohort studies with administrative datasets, focusing specifically on indicators of economic activity and education.  It also involved the creation of a new database to allow researchers to track the performance of schools more accurately and efficiently, helping to paint a clearer picture of education in England: the Consistent Schools Database. This links data on school performance, mergers, splits, closures and changes in school status, both forwards and backwards in time. It currently covers data from 1999 through to 2014. Its creation involved two stages: (i) making the Department of Education's School Level Database (and its predecessor, the Local Education Authority School Information Service) longitudinally consistent, and (ii) linking schools across years iteratively using a series of link variables to reduce unaccounted-for non-linkage. |
| Geographic Data | Enable the linkage of British cohort study data with geo-contextual data | ▫ BCS70 ▫ NCDS (With supplementary work on MCS and ALSPAC) | Chris Dibben, University of Edinburgh | ▫ NCDS sweep 3 (1974) geographic data: *http://doi.org/10.5255/UKDA-SN-8218-1*  ▫ NCDS sweep 4 (1981) geographic data: *http://doi.org/10.5255/UKDA-SN-8220-1*  ▫ NCDS sweep 5 (1991) geographic data: *http://doi.org/10.5255/UKDA-SN-8221-1*  ▫ BCS70: 10-year follow-up (1980) geographic data: *http://doi.org/10.5255/UKDA-SN-8211-2*  ▫ BCS70 16-year follow-up (1986) geographic data: *http://doi.org/10.5255/UKDA-SN-8212-2*  ▫ BCS70 26-year follow-up (1996) geographic data: *http://doi.org/10.5255/UKDA-SN-8213-2*  ▫ A review of resources for geographic variables: *https://www.closer.ac.uk/wp-content/uploads/CLOSER-resource-A-review-of-resources-for-geographical-variables.pdf* | The project explored how geographical variables can be linked to longitudinal data, and explored the types of data that are available. A report was produced to help researchers interested in how best to enrich their analyses with contextual information about the areas in which longitudinal study participants live. The team also worked to convert specific cohort members’ addresses into geographic coordinates in a non-identifiable manner to enable the study members’ data to be linked with this contextual geographic data.  Unit postcodes were sourced from addresses collected during interview and validated by the Centre for Longitudinal Studies' Cohort Maintenance Team. The validated unit postcode was then used to link the data to a broad range of higher level areal units, including standard statistical region, electoral wards and enumeration districts. Further detail is available in the user guides that accompany the geocoded data accessible through the UK Data Service. |
| Health Data - Hospital Episode Statistics | Conduct a general population survey on issues around consent to health data linkage and undertake a pilot of such linkage with the CLOSER studies and Hospital Episode Statistics | ▫ ALSPAC ▫ HCS ▫ UKHLS | Andy Boyd, University of Bristol | ▫ Understanding Hospital Episode Statistics (HES): *https://www.closer.ac.uk/wp-content/uploads/CLOSER-resource-Understanding-HES.pdf* | This research project focused on the value gained by linking study data to health data from the NHS. It explored the types of consent used in UKHLS, ALSPAC and HCS, with a focus on linkage between study data and Hospital Episode Statistics (HES). HES is a database containing details of all admissions, accident and emergency attendances and outpatient appointments at NHS hospitals in England. Researchers can now benefit from a CLOSER resource report to help them link longitudinal data to HES. This is available from the CLOSER website. |
| Linkage in the CLOSER Studies | Undertake a case study of cross-cohort analysis using primary care and education records to identify practical challenges faced in data linkage | ▫ ALSPAC ▫ NCDS ▫ Whitehall II ▫ UK Biobank ▫ Born in Bradford | Andy Boyd, University of Bristol | ▫ NHS Digital - Precedents set: *https://www.closer.ac.uk/wp-content/uploads/CLOSER-resource-NHS-Digital.pdf*  ▫ NHS Number and their management systems: *https://www.closer.ac.uk/wp-content/uploads/CLOSER-resource-NHS-Numbers-and-their-management-systems.pdf* | This project focused on helping cohort and longitudinal studies overcome barriers to data linkage and highlighted ways of linking study data to routine health records, including Detailed discussions were held with key stakeholders such as NHS Digital and the studies. Guidance on data linkage have been disseminated through knowledge exchange workshops. CLOSER resource reports were also produced for researchers, offering guidance and methodology on linking longitudinal data to health records. This includes documentation on the precedents that have been set by successful data linkage applications to NHS Digital, the national information and technology partner to the UK health and social care system. |
